# Describing the scattering of keV protons through graphene

**DOI:** 10.3389/fchem.2023.1291065

**Published:** 2023-11-16

**Authors:** Jakob Bühler, Philippe Roncin, Christian Brand

**Affiliations:** ^1^ Department of Quantum Nanophysics, German Aerospace Center (DLR), Institute of Quantum Technologies, Ulm, Germany; ^2^ Institut des Sciences Moléculaires d’Orsay (ISMO), Centre national de la recherche scientifique (CNRS), University Paris-Sud, Université Paris-Saclay, Orsay, France

**Keywords:** graphene, scattering, protons, statistical averaging, 2D-materials, analytic method

## Abstract

Implementing two-dimensional materials in technological solutions requires fast, economic, and non-destructive tools to ensure efficient characterization. In this context, scattering of keV protons through free-standing graphene was proposed as an analytical tool. Here, we critically evaluate the predicted effects using classical simulations including a description of the lattice’s thermal motion and the membrane corrugation via statistical averaging. Our study shows that the zero-point motion of the lattice atoms alone leads to considerable broadening of the signal that is not properly described by thermal averaging of the interaction potential. In combination with the non-negligible probability for introducing defects, it limits the prospect of proton scattering at 5 keV as an analytic tool.

## 1 Introduction

There are numerous experimental techniques available to study and analyze two-dimensional (2D) materials. Most commonly Raman spectroscopy [Ferrari et al. (2006); Tan (2019); Saito et al. (2016)], electron microscopy [Meyer et al. (2007); Li et al. (2018); Chen et al. (2020)], and scanning probe microscopy [Novoselov et al. (2004); Zhang et al. (2018); Marchini et al. (2007)] are employed. These are complemented by other methods, such as diffraction and scattering of massive particles at the membranes [Woznica et al. (2015); Brand et al. (2015); Brand et al. (2019); Al Taleb et al. (2015); Debiossac et al. (2016); Borca et al. (2010); Maccariello et al. (2015); Tamtögl et al. (2015); Anemone et al. (2016); Benedek et al. (2021); Tømterud et al. (2022); Al Taleb and Farías (2016); Tamtögl et al. (2021); Sacchi and Tamtögl (2023); Bahn et al. (2017); Jiang et al. (2019); Jiang et al. (2021)]. They yield valuable insights into the large-scale structure of the membrane, interaction potentials, and low-energy excitations. In such measurements, often atomic beams are used. As the interaction energy is typically in the meV-regime, this approach is completely non-destructive [Al Taleb et al. (2015)]. It sheds light on the particle-membrane interaction in the low-energy regime [Zugarramurdi et al. (2015); Debiossac et al. (2016)] and is used to assess the interaction of graphene with various materials [Borca et al. (2010); Maccariello et al. (2015); Tamtögl et al. (2015); Anemone et al. (2016)]. If the membrane is weakly bound to the substrate, intrinsic properties of materials can be assessed, such as electron-phonon couplings [Benedek et al. (2021)] and the membrane’s bending rigidity [Tømterud et al. (2022); Al Taleb and Farías (2016)]. Moreover, it is also possible to study the dynamics of adsorbates on membranes [Tamtögl et al. (2021); Sacchi and Tamtögl (2023); Bahn et al. (2017)], including the creation of transient molecular bonds [Jiang et al. (2019); Jiang et al. (2021)].

In this context transmission of protons at 5 keV through graphene has been proposed as an analytic tool [Ćosić et al. (2018); Ćosić et al. (2019); Hadžijojić et al. (2021); Ćosić et al. (2021)]. Describing the interaction of protons with the membrane via a thermally averaged potential, two rainbow features are predicted: an “inner” and an “outer” one. The inner rainbow is present at a few mrad and arises from trajectories close to center of the hexagon. The outer one stems from a maximum in the deflection function close to a carbon atom, leading to a signal at around 170 mrad [Ćosić et al. (2018)]. The authors argue that in combination these give very detailed insights into the internal temperature [Ćosić et al. (2019)] and the interaction potential [Ćosić et al. (2021)], the defect concentration [Hadžijojić et al. (2021)] as well as the membrane’s orientation and inclination [Ćosić et al. (2018); Ćosić et al. (2021)]. However, there are several conceptual issues with the underlying theoretical description. For instance, even when the protons hit a nucleus head-on they are always transmitted.

In this publication, we provide a description of the scattering process based on classical trajectories. We incorporate thermal effects by statistical averaging over displaced carbon atoms for each individual scattering event. Additionally, we account for the membrane’s corrugation as well as the experimental resolution and quantify their impact on the pattern at the detector. Based on this, we evaluate the predictions from the literature [Ćosić et al. (2018); Ćosić et al. (2019); Hadžijojić et al. (2021); Ćosić et al. (2021)] and test whether classical scattering through membranes can be used as an analytic tool. In our analysis we find that the predicted outer rainbow is an artifact from an improper description of the scattering process. For the inner rainbow already the zero-point motion of the membrane atoms leads to a significant broadening of the signal, obscuring part of the details. Together with the fact that protons at 5 keV have a non-negligible cross-section for displacing atoms in graphene [Shi et al. (2019)], this limits their use as an analytic tool for the study of 2D materials.

## 2 Theory and methods

**FIGURE 1:**
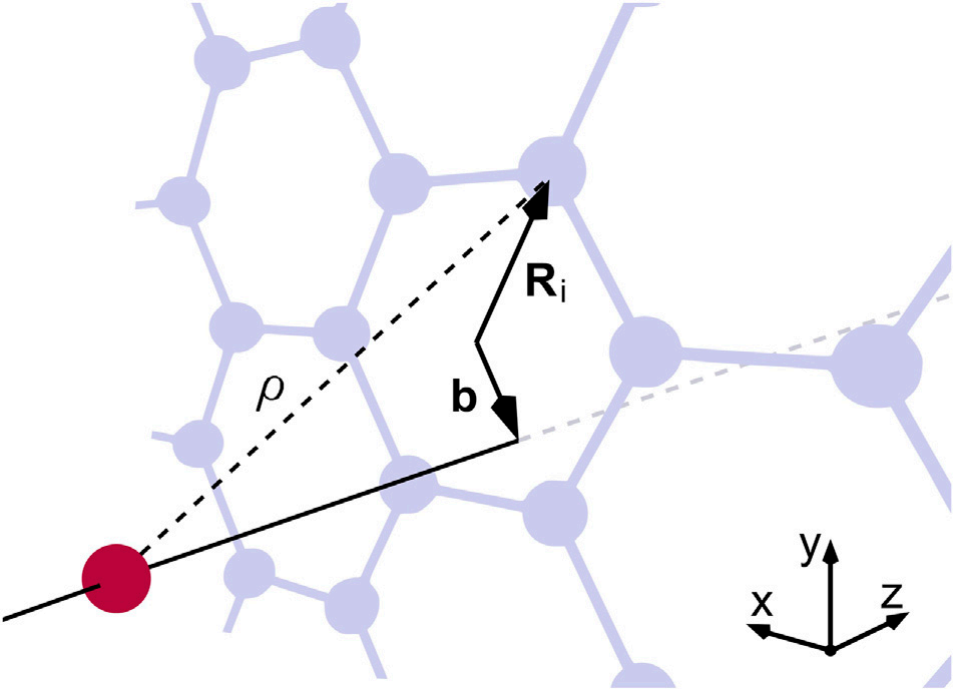
Schematic of a proton (red) impinging on a graphene lattice (grey) at right angles. The interaction with an individual carbon atom depends on *ρ* and the in-plane distance *r*, which is the difference between the impact parameter **b** and the position vector **R**_**i**_ of the atom.

To capture the behavior described previously in the literature [Ćosić et al. (2018); Ćosić et al. (2019); Hadžijojić et al. (2021)], we consider the transmission of protons with a kinetic energy of 5 keV through single-layer graphene. These particles propagate along the *z*-direction with velocity *v*_*z*_ until they interact with the 2D membrane where they are scattered, as shown in Figure 1. To describe the interaction of the collision partners, we use the ZBL potential [Ziegler and Biersack (1985); Ziegler et al. (1983)]

$$V^{\text{ZBL}}\left(\rho \right)=\frac{Z_{\text{H}}\;Z_{\text{C}}\;e^{2}}{4\pi \varepsilon_{0}}\cdot \frac{1}{\rho }\underset{n}{\sum }\alpha_{n}\exp\left[-\beta_{n}\frac{\rho }{a}\right].$$ (1)

Here, *ρ* is the proton-carbon distance, *Z*_H_ and *Z*_C_ are the atomic numbers of hydrogen and carbon, *e* is the elementary charge, and *ɛ*_0_ is the vacuum permittivity. The screening radius *a* and the fitting parameters *α*_*n*_ and *β*_*n*_ account for the distance-dependent shielding of the nuclear Coulomb interaction by the electrons [Ziegler et al. (1983)].

For small scattering angles (*θ* ≪ 1 rad) the magnitude of the transferred transverse momentum *p*_⊥_ for the binary interaction between a proton and a lattice atom is

$$p_{⊥}\left(r\right)=-\frac{r}{v_{z}}\int_{-\infty }^{\infty }\frac{\text{d}V\left(\rho \right)}{\text{d}\rho }\frac{1}{\sqrt{z^{2}+r^{2}}}\;\text{d}z,$$ (2)

where *r* is the proton-carbon distance in the scattering plane and $\rho =\sqrt{z^{2}+r^{2}}$ . The total transverse momentum vector **p**_⊥,tot_ depends on the impact parameter **b** and is obtained by summing over all considered binary interactions [Lehtinen et al. (2010)]

$$p_{⊥,tot}\left(b\right)=\underset{i}{\sum }p_{⊥}\left(\left\|b-R_{i}\right\|\right)\frac{b-R_{i}}{\left\|b-R_{i}\right\|}.$$ (3)

The deflection angle ***θ*** = (*θ*_*x*_, *θ*_*y*_) is obtained within the small-angle approximation

$$\theta =\frac{p_{⊥,tot.}}{p_{z}}.$$ (4)

While here the protons are always scattered through a single, central carbon ring, we added six additional hexagons around the central one to form a coronene-like structure. Furthermore, we restrict the integration in Eq. 2 to ±1 nm around the scattering plane (see Supplementary Material). In this description we treat the protons as classical particles and neglect the wave-nature of the protons. This is justified by the significant experimental challenges that ions pose to matter-wave diffraction, which have not yet been overcome.

To account for the finite angular resolution, we assume a 2D-Gaussian distribution of incoming angles with a variable standard deviation. For each scattering event, a random angle was chosen from the distribution and added to the calculated scattering angle. Also, the natural corrugation of free-standing single-layer graphene, that is, its three-dimensional waviness was included [Meyer et al. (2007)]. When protons scatter at a corrugated membrane, the effective scattering geometry corresponds to the projection of the graphene sheet onto the *xy*-plane. Here, we consider a root-mean-squared inclination of 75.7 mrad, corresponding to the experimental value reported for exfoliated graphene [Singh et al. (2022)].

### 2.1 Modelling thermal motion

A proton with an energy of 5 keV moves at 979 km/s, traversing the interaction zone of 2 nm within 2 fs. This has to be compared to the frequency range of phonons in graphene, which reaches up to 50 THz [Yang et al. (2021)]. So, even at the highest phonon mode, atoms undergo only 0.1 vibrations while a proton is within the interaction zone. Hence, the movement of the atoms can be approximated as quasi-static [Pfandzelter et al. (2001)]. However, each proton encounters a different scattering geometry and one has to average over many different scattering geometries to obtain a realistic picture. Using the Debye model for the density of states [Kittel (2018)] and describing each atom as a harmonic oscillator around its lattice site [Cohen-Tannoudji et al. (1991)], the mean squared displacement *σ* ^2^ can be expressed as a function of membrane temperature *T* [Chen and Yang (2007)]

$$\sigma^{2}=\frac{3\hbar^{2}}{m_{\text{C}}k_{\text{B}}\Theta_{\text{D}}}\left[\frac{T}{\Theta_{\text{D}}}D_{1}\left(\frac{\Theta_{\text{D}}}{T}\right)+\frac{1}{4}\right].$$ (5)

Here *m*_C_ is the mass of a carbon atom, *k*_B_ is the Boltzmann constant, *Θ*_D_ = 2100 K the in-plane Debye temperature of graphene [Tohei et al. (2006); Pop et al. (2012)], and *D*_1_ the first Debye function. We use the same model to describe the zero-point motion of the lattice atoms. For *T* close to 0 K, Eq. 5 simplifies to

$$\sigma_{0}^{2}=\frac{3}{4}\frac{\hbar^{2}}{m_{\text{C}}k_{\text{B}}\Theta_{\text{D}}}$$ (6)

resulting in an in-plane displacement for single-layer graphene of $\sigma_{0}^{2}=1.4\times 10^{-5}$ nm^2^. In this study, we assume an uncorrelated 2D normal distribution for the in-plane displacement of each atom. Out-of-plane displacements have not been included, as they have no effect on the signal within this description.

## 3 Results

**FIGURE 2:**
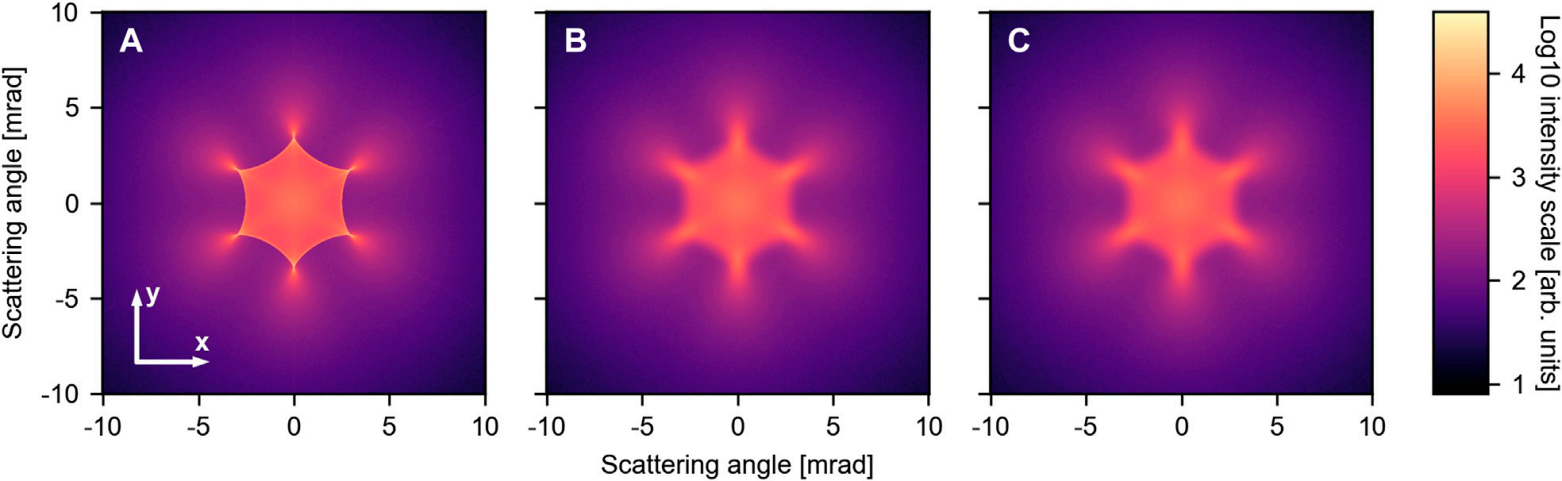
Scattering simulations including different effects: **(A)** Flat membrane with atoms fixed at their equilibrium position, **(B)** flat membrane exhibiting zero-point motion, and **(C)** corrugated membrane with a root mean square (rms) inclination angle of 75.7 mrad at *T* =300 K. In **(C)** we further included an angular resolution of 100 µrad. All intensities are plotted on a logarithmic scale.

We first consider a perfectly collimated beam impinging on a flat membrane where the position of all carbon atoms is fixed at their equilibrium position. While such a situation cannot be realized experimentally, the respective pattern shown in Figure 2A may act as a point of reference for the latter simulations. It exhibits a six-fold symmetry, mirroring the honeycomb structure of graphene. Most intensity is scattered at angles below 7.5 mrad and we observe rather sharp features. These can be traced back to rainbow scattering originating from a maximum in the deflection angle close to the center of the hexagon. For larger scattering angles, the signal continuously decreases in intensity with no discernible features, as shown in the Supplementary Material. Thus, we do not observe the outer rainbow feature reported previously in the literature [Ćosić et al. (2018); Ćosić et al. (2019); Hadžijojić et al. (2021)]. We can, however, artificially reproduce these features by thermally averaging the interaction potential (Supplementary Material).

**FIGURE 3:**
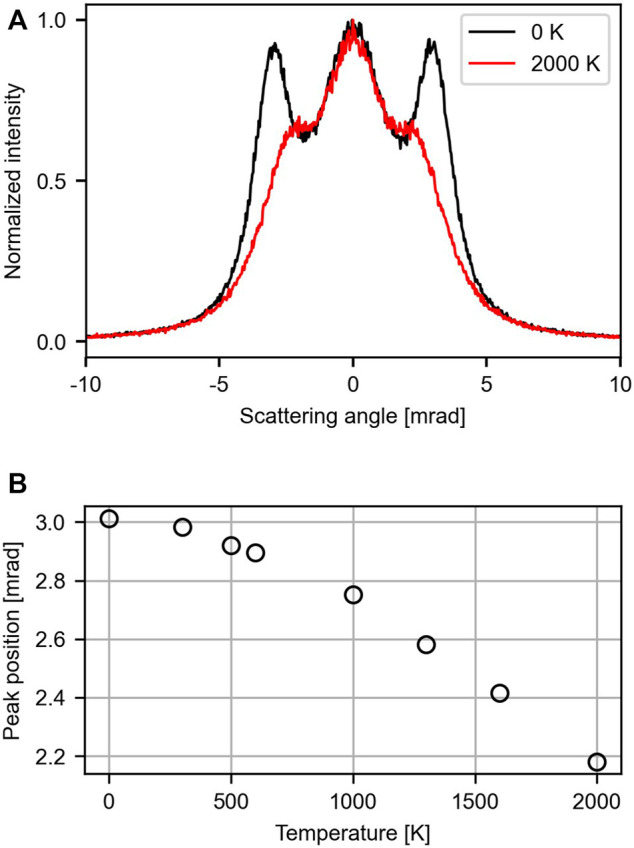
Influence of the membrane temperature on the scattering pattern. Here the effect of the membrane’s corrugation and the angular resolution was neglected. **(A)** Line traces through the *y*-axis shown for 0 K (black) and 2000 K (red). **(B)** Dependence of the side lobe’s position on temperature between 0 and 2000 K.

Including the lattice atoms zero-point motion already leads to a significant broadening of the rainbow lines, as shown in Figure 2B. While the general shape remains unaffected, the fine features are washed out. To study the temperature-dependence, we extended the simulations to a range between 0 and 2000 K. The line plots through the center of the pattern along the *y*-axis for 0 and 2000 K are shown in Figure 3A. The data for the other temperatures and the traces through the *x*-axis can be found in the Supplementary Material. In general, the plots exhibit just a few features: a central lobe and the rainbow peaks located at ≈3.0 mrad. With increasing temperature these peaks become less prominent and shift to smaller angles. At 2000 K they are reduced to shoulders located at *θ* ≈ 2.2 mrad. The dependence of the peak position on the temperature is shown in Figure 3B.

**FIGURE 4:**
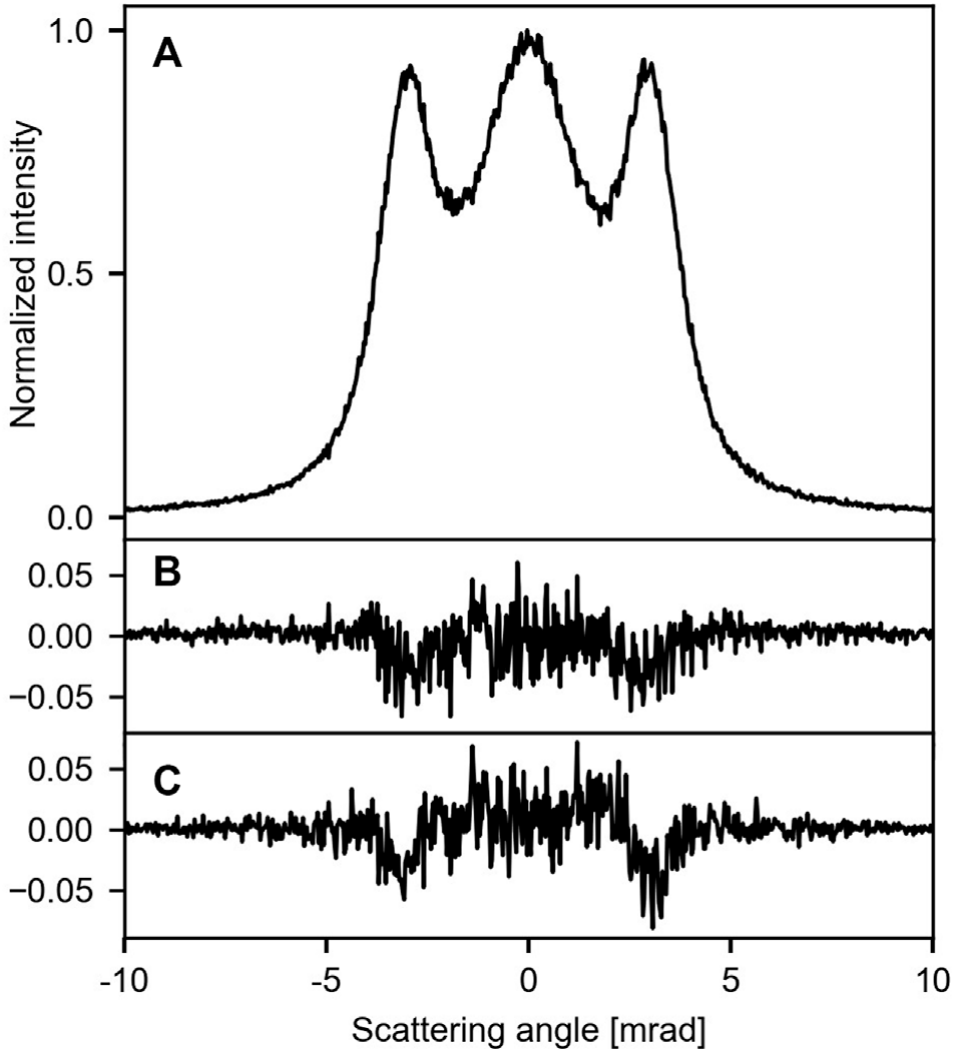
Influence of the corrugation and the angular resolution on the signal at 0 K (traces along the *y*-axis through the center): **(A)** flat membrane and infinite angular resolution, **(B)** corrugated membrane with a rms inclination of 75.5 mrad, **(C)** flat membrane and an angular resolution of 100 µrad. For **(B, C)** the difference to trace **(A)** is shown.

Both the membrane’s corrugation and the angular resolution may lead to further broadening of the signal at the detector. This is shown in Figure 4. In comparison to a flat membrane (Figure 4A), the effect of the corrugation [Singh et al. (2022)] on the pattern is on the percentage level and thus challenging to resolve (Figure 4B). The same is true for an angular resolution of *ϕ* = 100 µrad (Figure 4C). Combining these two effects thus yields a pattern, which is virtually identical to the flat membrane. This is illustrated in Figures 2B, C for *T* = 300 K. Degrading the angular resolution leads to a decrease in the relative intensity of the side lobes. Furthermore, their position is shifted to smaller angles (Supplementary Material), resembling the effect of increasing temperature.

## 4 Discussion

Classically, rainbow scattering occurs at the extrema of the scattering function, which maps the impact parameter to a scattering angle *θ*. Usually, this is the case at inflection points of the scattering potential and results in a sharp intensity maximum at the corresponding scattering angle. Thus, the shape and the position of the rainbow pattern contain information about the interaction potential between the membrane and the protons.

Previous calculations of proton transmission through graphene used thermal averaging of the potential to describe the interaction [Ćosić et al. (2018)]. In that model, the charge of the C nucleus is spread over a volume $\propto \sigma_{0}^{3}$ , replacing the Coulombic singularity at *r* = 0 by a Gaussian. Its inflection point produces a second outer rainbow, which was predicted to be sensitive to temperature and the orientation of the membrane [Ćosić et al. (2018); Ćosić et al. (2019); Ćosić et al. (2021); Hadžijojić et al. (2021)]. However, it also entails that protons are transmitted during head-on collisions.

Thermal averaging of the interaction potential was introduced in channeling of high-energy ions through crystals [Krause et al. (1986)] and is still used to investigate possible bias in the detection of dark matter [Bozorgnia et al. (2010)]. During channeling the ion trajectory oscillates within the potential originating from the atomic strings. Thermal averaging of the potential along the channel is the simplest model to describe these complex oscillations and preserve the axial symmetry. However, this approach is limited and the effect of crystal atoms protruding into the channel requires a statistical evaluation [Andersen and Feldman (1970)]. In the case of 2D materials the thickness is orders of magnitude smaller than the typical distance between oscillations in the axial potential. This suggests that there are not enough interactions along the trajectory to justify an averaged potential.

We start the evaluation of the predicted effects by addressing the impact of temperature on the scattering pattern. As illustrated in Figure 3B, the peak position of the side lobes changes by only 0.12 mrad when going from 0 to 600 K. To realize a temperature resolution of 100 K (500–600 K), a shift in peak position relative to the peak width of only 4% has to be resolved, which we consider to be challenging. This has to be compared to Raman spectroscopy and surface diffraction where a temperature difference of about 60 K can be resolved [Calizo et al. (2009); Pan et al. (2022)]. Above 1000 K the impact of temperature becomes more pronounced, but the intensity of the side lobes deteriorates, making it harder to determine their position accurately. In general, such issues can be mended by a thorough characterization of the setup and long integration times. However, in the current situation we face two fundamental limitations. First, protons at 5 keV create a single vacancy in graphene with a probability 2 × 10^−3^ [Shi et al. (2019)]. Thus, the membrane is destroyed continuously during the measurement process. Second, the constant stream of protons colliding with the membrane leads to artificial heating. This suggests that scattering of protons at 5 keV provides only limited insights into the membrane’s temperature.

The large-scale corrugation of the membrane has only a minor effect on the scattering pattern. Thus, stretching the membrane as discussed in Nicholl et al. (2017); Nicholl et al. (2015) does not seem necessary. Regarding angular resolution, we observe that increasing the value of *ϕ* resembles the effect of increasing temperature (see Supplementary Material): at 400 µrad the intensity of the rainbow peaks is comparable to that at *T* = 2000 K, cf. Figure 3. The optimal resolution to study scattering of protons at 5 keV is around *ϕ* = 100 µrad. It can be easily realized experimentally and the resulting pattern (Figure 2C) is virtually indistinguishable from the one with a perfectly collimated beam (Figure 2B). This allows to capture all essential details of the pattern.

So far, we have considered a perfect crystal in a single orientation. This is motivated by advances in the synthesis of graphene [Chen et al. (2013); Gao et al. (2012); Wu et al. (2013); Yan et al. (2014)]. However, irradiating such a sample with protons at 5 keV leads to substantial damage. If we multiply the probability to introduce defects with the 1.6 × 10^8^ scattered protons required to create an image in Figure 2, we end up with 3.2 × 10^5^ additional generated vacancies. Thus, using this method to study defects and assess their concentration is at least questionable. Extracting information from a poly-crystalline sample with grain boundaries is even more challenging. Here, several lattices with different orientations will contribute, further obscuring the image. In the extreme case of small grain size one would expect a circularly symmetrical image. However, if the experiment is restricted to low doses, it should be possible to extract some information on the interaction potential from the position of the rainbow peaks before the membrane is damaged too much. The same applies to orientation of the membrane, which is encoded in the scattering pattern at least for samples with one predominant crystal orientation.

## 5 Summary and outlook

In summary, we have investigated classical scattering of protons through graphene. Including the temperature of the lattice by displacing the lattice atoms for each scattering event, we could show that the outer rainbow previously described in the literature is an artifact. In contrast to that, statistical averaging performed here quantitatively indicates the maximum level of detail that can be observed in an experiment. Regarding thermometry, we observe only a weak dependence of the peak positions on temperature, which might additionally be obscured by the angular resolution and the artificial heating due to the colliding protons. Based on this, we cannot confirm the predicted high sensitivity regarding temperature based on the contribution of in-plane and out-of-plane motion.

If the dose is restricted, it should be possible to extract some information on the interaction potential and the orientation of the membrane. The possibility to study defects seems unrealistic as the method has a non-negligible probability for inducing defects itself. This limits the applicability of the proposed method as an analytical tool as the membrane is always changed during the analysis.

To avoid beam damage and turn proton scattering into a useful technique, the interaction energy has to be reduced below the damage threshold, which is predicted around 80 eV [Brand et al. (2019)]. In turn, this opens new vistas to study the interaction of protons with the membrane. On the one hand, neutralization will be more prominent at these energies [Kononov and Schleife (2021)], bringing energy- and angle-resolved neutralization studies within reach. On the other hand, exchanging protons by neutral hydrogen atoms facilitates matter-wave diffraction [Brand et al. (2019)]. In this case the level of detail is expected to be much higher, allowing to study elastic and inelastic interactions in detail.

## Data Availability

The raw data supporting the conclusion of this article will be made available by the authors, without undue reservation.
