# Supplementary material for: Describing the scattering of keV protons through graphene
Source: Front Chem. 2023 Nov 16;11:1291065. doi: 10.3389/fchem.2023.1291065 (PMC10687178; doi:10.3389/fchem.2023.1291065)
Supplement: Supplementary file 1 [file DataSheet1.PDF]

Supplementary Material for describing the scattering of keV protons through graphene

1 LARGE SCATTERING ANGLES

To illustrate the behaviour at large scattering angles, simulations up to 300 mrad were performed. Here, only the magnitude of the scattering angle was binned while the direction of the total momentum transfer was disregarded. In Fig.S1 it is shown that for statistical averaging no additional features appear up to an angle of 300 mrad. When replicating the thermal averaging model [Ćosić et al. (2018)], we observe a secondary rainbow feature at about 180 mrad for a temperature of 0 K.

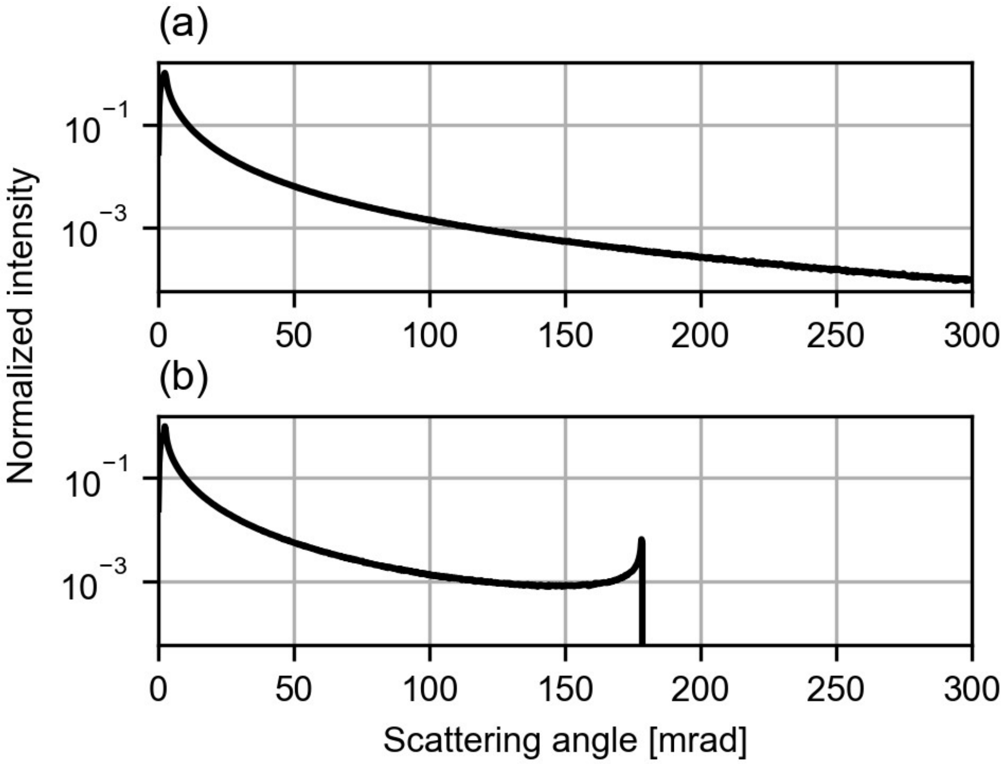

Figure S1. Counts integrated over the full polar angle up to large scattering angles. Membranes at 0 K modeled in two different ways: a) statistical averaging b) thermally averaged potential.

2 INTEGRATION RANGE

As the effective range of the binary interaction potential is on the order of dozens of picometers, we use an integration range of ± 1 nm to calculate the value of the deflection integral to a sufficient accuracy. We tested this assumption by comparing scattering patterns at a frozen lattice with an integration range of ± 1 nm and $\pm\infty$. As can be seen in Fig. S2, there is no discernible difference in the results besides the shot noise.

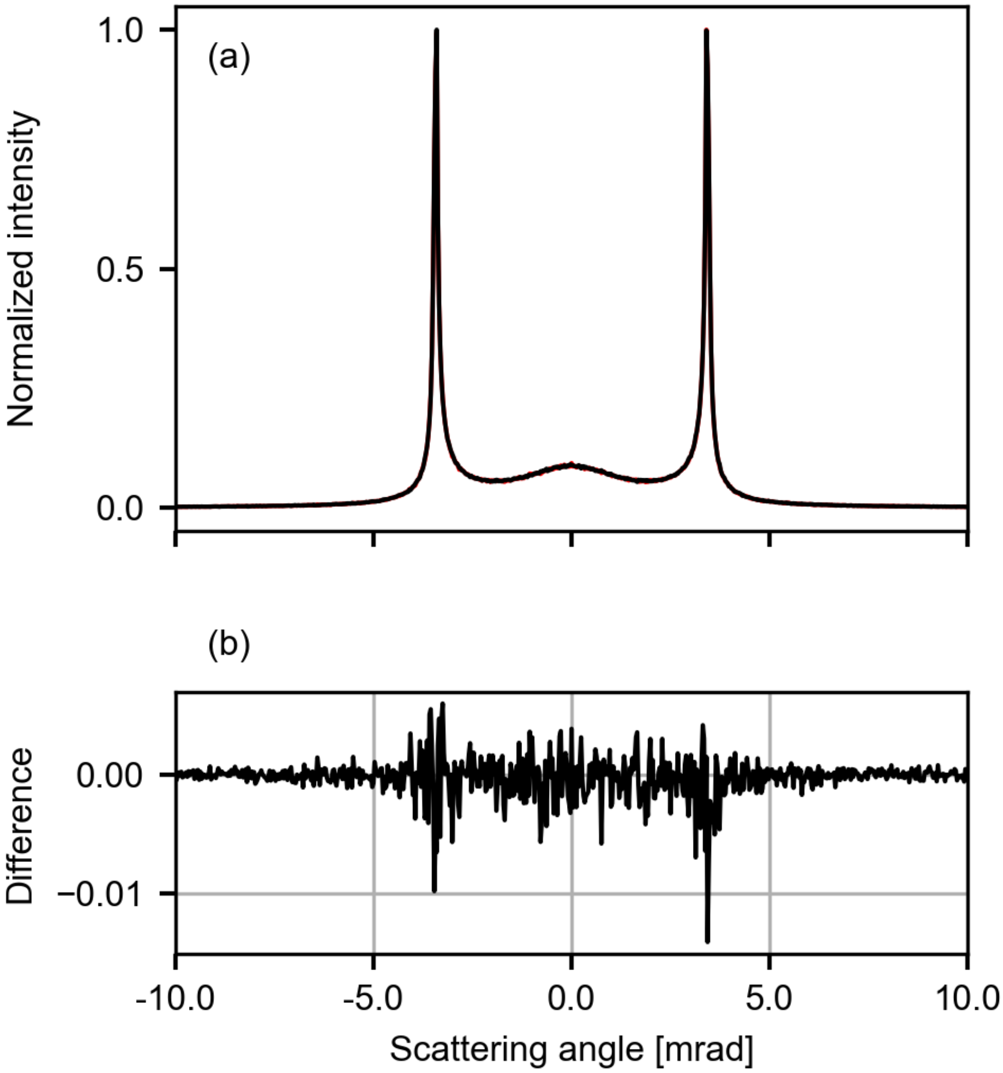

Figure S2. (a) Line traces along the y-axis through the central feature of scattering patterns with the membrane atoms frozen at their equilibrium positions. (red) Integration over ± 1 nm (black) Integration over $\pm\infty$. The lines are indistinguishable except for shot noise. (b) Difference of the line traces shown in (a).

3 TEMPERATURE DEPENDENCE

The scattering pattern at a hexagonal lattice has two distinct types of symmetry axes. In the main paper we showed only line traces through the y-axis, as it exhibits more distinct features. In Fig.S3 the line traces through both axes for seven temperatures between 0 and 2000 K are shown.

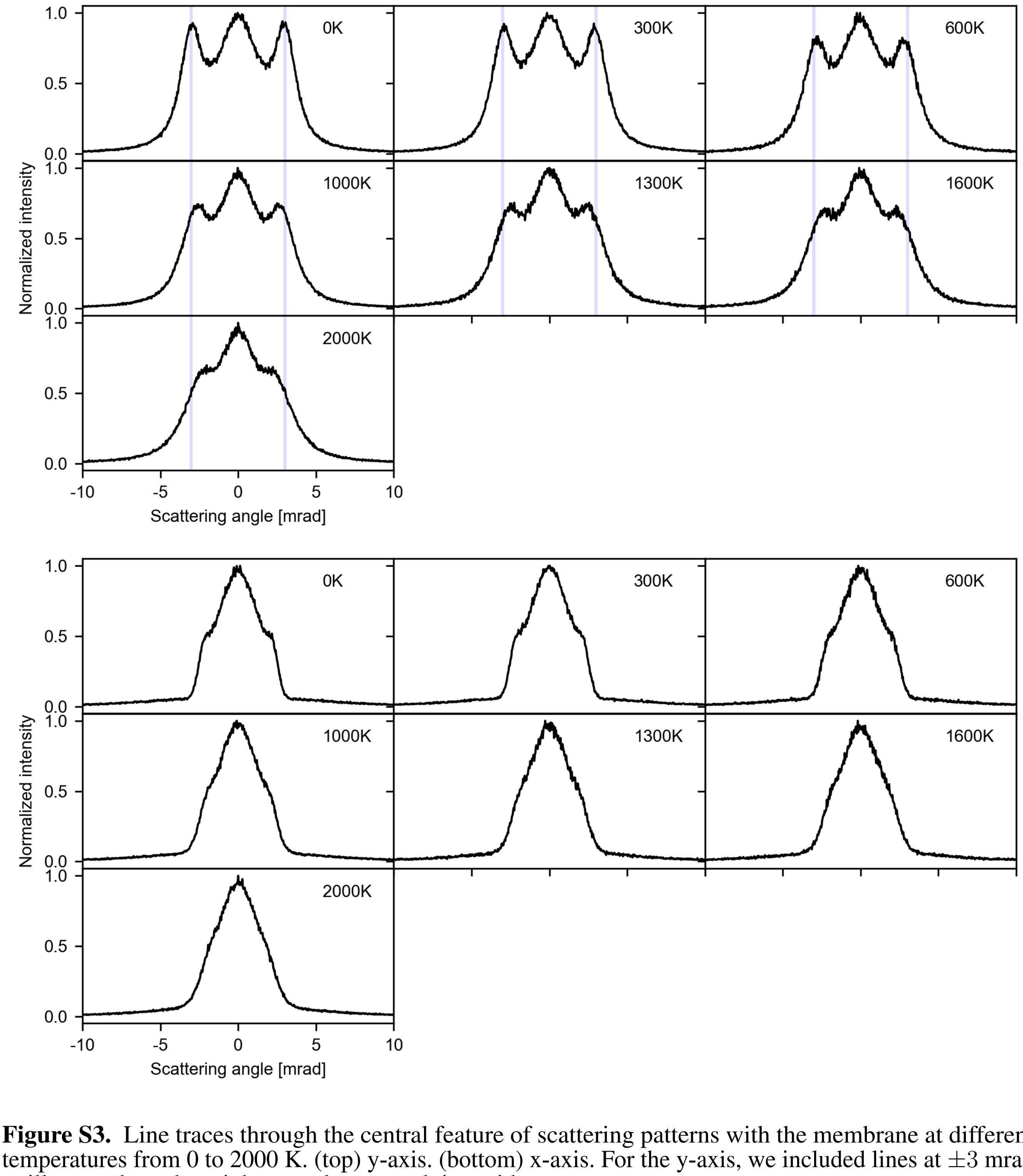

Figure S3. Line traces through the central feature of scattering patterns with the membrane at different temperatures from 0 to 2000 K. (top) y-axis. (bottom) x-axis. For the y-axis, we included lines at ± 3 mrad to illustrate how the rainbow peaks are evolving with temperature.

4 COLLIMATION

Angular resolution has a strong influence on the level of detail, which can be resolved in the experiment. As can be seen in Fig. S4 the effect of broader beam collimation is very similar to the effect of higher temperature.

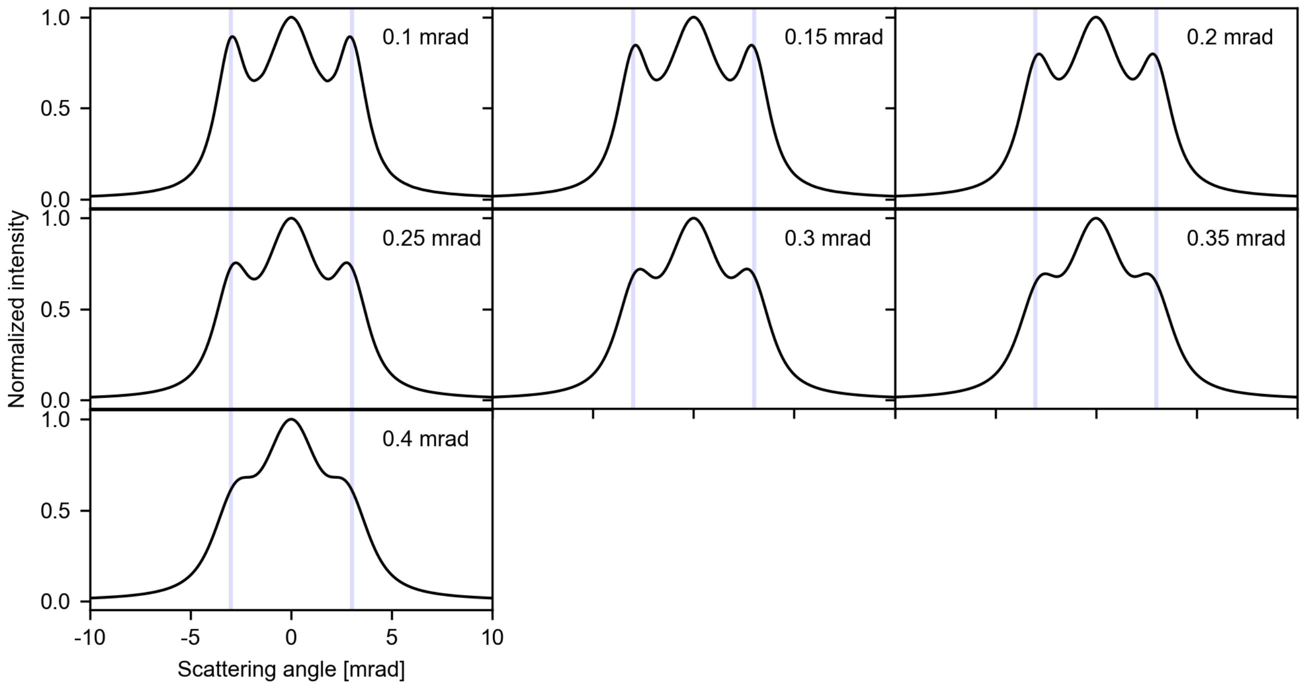

Figure S4. Line traces through the central feature of scattering patterns with the membrane at 0 K. For each image the pattern was convoluted with a kernel of differing standard deviation in order to emulate the effect of beam collimation.

REFERENCES

Ćosić, M., Petrović, S., and Nešković, N. (2018). The forward rainbow scattering of low energy protons by a graphene sheet. *Nuclear Instruments and Methods in Physics Research Section B* 422, 54–62
